# Development of Pd/TiO_2_ Porous Layers by Pulsed Laser Deposition for Surface Acoustic Wave H_2_ Gas Sensor

**DOI:** 10.3390/nano10040760

**Published:** 2020-04-15

**Authors:** Izabela Constantinoiu, Cristian Viespe

**Affiliations:** National Institute for Laser, Plasma and Radiation Physics, Laser Department, Atomistilor #409, 077125 Magurele, Romania; izabela.constantinoiu@inflpr.ro

**Keywords:** pulsed laser deposition (PLD), surface acoustic wave (SAW), hydrogen sensor, porous morphology, bilayer films, Pd/TiO_2_, titanium dioxide, palladium

## Abstract

The influence of sensitive porous films obtained by pulsed laser deposition (PLD) on the response of surface acoustic wave (SAW) sensors on hydrogen at room temperature (RT) was studied. Monolayer films of TiO_2_ and bilayer films of Pd/TiO_2_ were deposited on the quartz substrates of SAW sensors. By varying the oxygen and argon pressure in the PLD deposition chamber, different morphologies of the sensitive films were obtained, which were analyzed based on scanning electron microscopy (SEM) images. SAW sensors were realized with different porosity degrees, and these were tested at different hydrogen concentrations. It has been confirmed that the high porosity of the film and the bilayer structure leads to a higher frequency shift and allow the possibility to make tests at lower concentrations. Thus, the best sensor, Pd-1500/TiO_2_-600, with the deposition pressure of 600 mTorr for TiO_2_ and 1500 mTorr for Pd, had a frequency shift of 1.8 kHz at 2% hydrogen concentration, a sensitivity of 0.10 Hz/ppm and a limit of detection (LOD) of 1210 ppm. SAW sensors based on such porous films allow the detection of hydrogen but also of other gases at RT, and by PLD method such sensitive porous and nanostructured films can be easily developed.

## 1. Introduction

The final properties of the materials are strongly influenced by their morphology. Thus, it is very important for each type of application to study an optimum morphology, so as to obtain its best results. This is also a significant aspect in the field of gas sensors. Besides stability, in this domain, a large area of interactions between the sensitive material and gas is desired. In other words, nanostructured porous materials are ideal for obtaining the highest sensitivity for gas sensors because they possess high surface to volume ratio, fast charge diffusion and large penetration depth [1,2].

Pulsed laser deposition (PLD) [3], radio frequency (RF) magnetron sputtering deposition [4], sol-gel method [5], chemical vapor deposition [6], etc. are synthesis methods by which nanostructured materials with different morphologies can be obtained. Among various techniques, PLD has the advantages of high deposition rate, maintaining the stoichiometry of the target, wide choice of materials and a relatively high reproducibility [7,8]. This technique has some unique features: the control of stoichiometry, the possibility to use thermally stable substrates, the capability to grow nanostructures in the presence of a gas [8]. These advantages come from the ability to control the parameters as laser power, pulse frequency, substrate temperature, rate deposition, pressure deposition, the distance between target and substrate, etc. [7,9]. In order to obtain various morphology and properties of the materials through the PLD technique, the deposition pressure is an important parameter influencing this feature [3,10,11]. As the depositing pressure increases, the porosity of the material also increases [12]. 

Surface acoustic wave (SAW) gas sensors operate on the principle of turning an electrical input signal into a mechanical wave [13]. The occurrence of gas molecules at the sensor sensitive film, leads to a perturbation of surface acoustic wave propagation. This phenomenon is due to changes in the mass or acoustoelectric properties of the sensitive layer in the presence of the gas [14]. This type of sensors has attracted attention through their features as small size, low cost, ease of fabrication, fast response, remarkable sensitivity, satisfactory stability, wireless operation [15,16]. SAW sensors have been developed for the detection of hydrogen [15,17] volatile organic compounds [18], ammonia [16], hydrogen sulfide [1], explosives [19], toxic gases [20], etc. 

Because hydrogen is a potential candidate to replace fossil fuel for sustainable transportation by providing clean, convenient, reliable, customer and affordable energy [21], the development of hydrogen sensors is required. Whereas, hydrogen is an odorless, colorless and vastly combustible gas: flammable between 4%–75% in air, with high diffusion coefficient in air (0.60 cm^2^/s), and low ignition energy (0.002 mJ), the failure to detect it in time can lead to an explosion [22,23]. Over time, several types of hydrogen sensing sensors have been developed, including chemiresistive gas sensors [24], SAW sensors [12], metal-oxide semiconductor gas sensors [23]. Different types of sensing materials have been used for hydrogen detection: metal oxide thin films (ZnO, TiO_2_, WO_3_, SnO_2_, In_2_O_3_) [25,26], metal thin films (Pd, Pt) [27], multilayer of metal oxides and metals (Pd/WO_3_, Pd/V_2_O_5_, Pd/SnO_2_, Pd/ZnO) [12,23,28,29], composite materials [30], nanowires [31]. Some of these types of sensors have the ability to detect hydrogen at temperatures above 200 °C, which is inappropriate for hydrogen detection because the risk of the explosion becomes higher especially at high hydrogen concentrations [30]. 

In this paper, we will focus on how the morphology of two of these commonly used materials in the field of hydrogen sensors, namely TiO_2_ and Pd, influence the response of SAW sensors at different hydrogen concentrations at room temperature (RT). 

TiO_2_ is one of the most popular hydrogen sensing materials, with remarkable results when it was used with different metals. Up to now, for detection at RT, TiO_2_ based sensors have results by surface modification with metals like Pd [32,33]. Because of the capacity to absorb large quantities of hydrogen (0.56 wt % at 293 K) and its low activation energy, Pd is widely used in hydrogen gas sensors [34]. It works as a catalyst by dissociating the hydrogen molecules in hydrogen atoms and by minimizing the activation energy between the surfaces of metal oxide and hydrogen gas [28]. 

For the detection at RT of hydrogen with SAW sensors, by the deposition method of PLD, sensitive films of TiO_2_ and Pd/TiO_2_ with different morphologies were developed. SAW sensors tests have demonstrated sensitivity at RT and the influence of porous morphology on improving sensor responses.

## 2. Materials and Methods 

### 2.1. Film Deposition and Characterization

For film deposition, a Nd-YAG laser (EKSPLA NL301HT, Ekspla, Vilnius, Lithuania) with an emission wavelength of 355 nm and a 5 ns pulse duration at a repetition rate of 10 Hz was used. The energy per pulse was about 71 mJ and by focusing the laser beam onto the target, an energy density of about 25 J/cm^2^ was obtained. 

The depositions were made on the quartz SAW sensor substrates. To limit the deposition only in the sensitive area of the sensor, we used a mask that protected the interdigital transducers (IDTs). The substrate and the target were placed parallel in the deposition chamber, and the target was controlled by computer x–y tables to ensure the ablation on a certain surface. The distance between the target and the substrate was 40 mm, and the deposition took place without heating the substrate. The depositions were made in the presence of two gases (oxygen and argon) whose pressure and gas flow were controlled through a system attached to the deposition facility. This system consists of a throttle valve (MKS 253B) controlled by a pressure controller (MKS 600) on a vane vacuum pump (Agilent Varian-DS602, Leini, Italy) and a mass-flow controller (MKS multigas 647) on the gas bottles. 

The sensitivity films for SAW sensors were deposited from two targets: TiO_2_ and Pd, in two forms: monolayer (only TiO_2_) and multilayer (Pd and TiO_2_). In addition to the type of film, the pressure of the gas in chamber deposition was also variated to establish the optimum morphology required in SAW sensors. Table 1 shows the deposition pressures used to achieve the six sensors developed in this work. As can be seen, for the three sensors with bilayers, the oxygen pressure of TiO_2_ deposition was maintained at 600 mTorr. This was determined after analyzing scanning electron microscopy (SEM) images and the results of the sensors tests at different hydrogen concentrations. 

The morphology of the sensitive layers and bilayers was studied by SEM (FEI QUANTA). 

### 2.2. Sensor Structure and Testing

The SAW sensors used are based on quartz substrates, with ST-X-cut and with a parallelogram geometry with 45° angle, to reduce the influence of the SAW unwanted reflections. The advantage of the quartz substrate is good stability at RT [12]. It is a delay line type sensor, having an oscillating frequency of ~69 MHz [35]. The dimensions of the sensor substrate are: 38 mm long, 10 mm wide, 0.5 mm thick. Using the photolithographic technique, the gold IDTs were deposited onto 10 nm thick chromium layers, which has the role of ensuring the gold adhesion to the quartz substrate. The configuration of the IDTs is ‘double-comb’ and consists of 50 straight pairs with 45.2 µm wavelength and 2500 µm acoustic aperture. The active surface of the sensor deposited by PLD between IDTs is 10 × 10 mm^2^. 

The set-up used for sensors tests is composed of a DHPVA-200 FEMTO amplifier (Messtechnik GmbH, Berlin, Germany) with a CNT-91 Pendullum frequency counter (Spectracom Corp, Rochester, NY, USA) connected to a computer software, Time View 3. 

**Figure 1:**
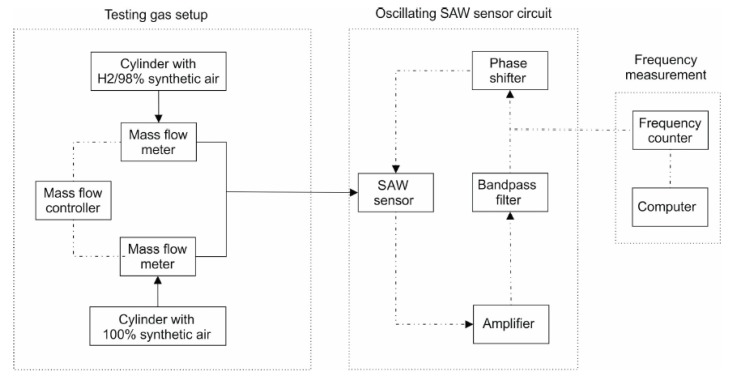
Experimental setup for surface acoustic wave (SAW) sensor frequency shift measurements for hydrogen detection [29].

The sensors were tested at different hydrogen concentrations. To obtain these concentrations, it was used mass flow controllers (Figure 1 [29]) to homogenize the hydrogen gas mixture (2%-H_2_ and 98% synthetic air) with pure synthetic air. The value of the total gas flow rate was maintained at 0.5 L/min in all determinations.

## 3. Results and Discussion

### 3.1. Film Morphology

**Figure 2:**
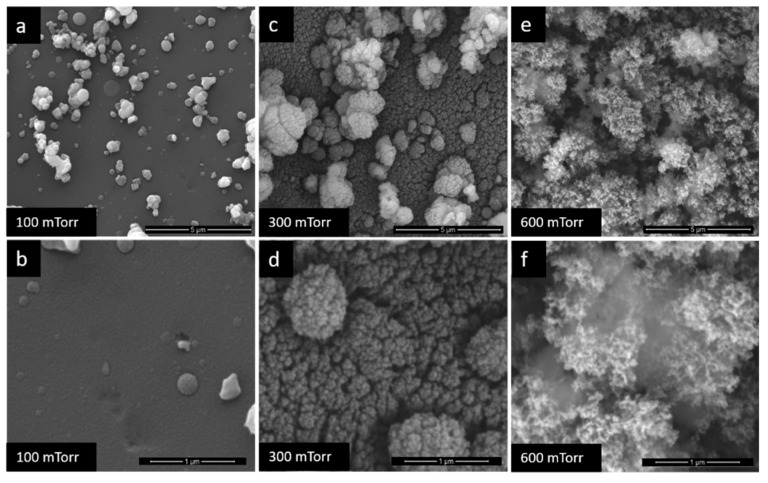
Scanning electron microscopy (SEM) images of TiO_2_ films at (**a**) and (**b**) −100 mTorr, (**c**) and (**d**) −300 mTorr, (**e**) and (**f**) −600 mTorr.

**Figure 3:**
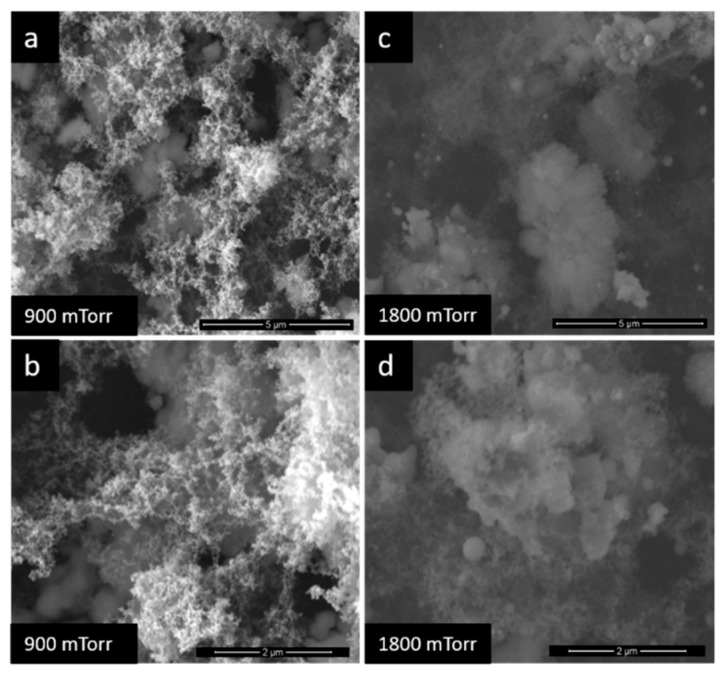
SEM images of TiO_2_ films at (**a**) and (**b**) −900 mTorr, (**c**) and (**d**) −1800 mTorr.

The different pressures under which the TiO_2_ films were deposited led to the obtaining of obviously different film morphologies, as it can be seen in Figure 2 and Figure 3. If at 100 mTorr the film is almost dense (Figure 2a,b), above 900 mTorr, the films become very porous with a tendency of exfoliation on the substrate (Figure 3). The explanation of this phenomenon lies in the delay effect produced on the species ablated by the collision between them and the gas molecules, which lead to the decrease of their kinetic energy and cluster nucleation [29,36]. Such porous morphology results in superior sensor results, even at RT determinations. 

Because at pressures above 900 mTorr the TiO_2_ films are no longer stable on the surfaces of the substrates, for the sensor’s sensitive films only the first three deposition pressures were chosen: 100 mTorr, 300 mTorr and 600 mTorr. Considering that at 100 mTorr, the film is not porous and for the other two pressures there are different degrees of porosity, it will be possible to analyses through the sensor’s results how this parameter influences the improvement of the sensitivity of the hydrogen SAW sensors at RT. 

**Figure 4:**
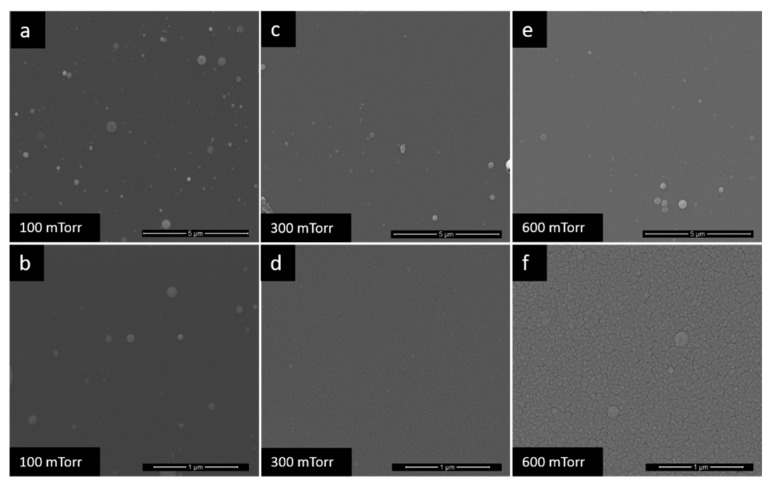
SEM images of the Pd films at (**a**) and (**b**) −100 mTorr, (**c**) and (**d**) −300 mTorr, (**e**) and (**f**) −600 mTorr.

**Figure 5:**
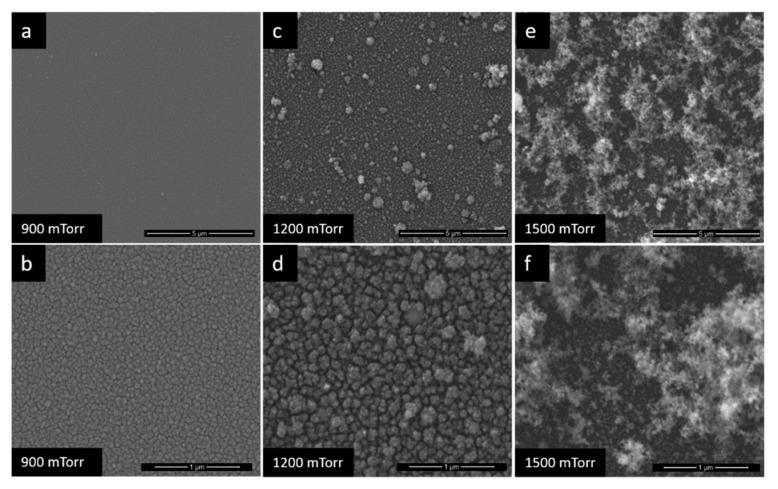
SEM images of the Pd films at (**a**) and (**b**) −900 mTorr, (**c**) and (**d**) −1200 mTorr, (**e**) and (**f**) −1500 mTorr.

The behavior of Pd at different pressures of PLD deposition was analyzed, in order to obtain multilayer films. The study that determined how the variation of gas pressure influences the morphology of Pd films was performed over a wider range of pressures: 100–2700 mTorr. As can be seen in the SEM images (Figure 4 and Figure 5), at 100 mTorr (Figure 4a,b), the film is dense. Going to 300 mTorr (Figure 4c,d), small cracks appear on the surface of the film and increasing the pressure to 600 mTorr, (Figure 4e,f) respectively 900 mTorr (Figure 5a,b), the size of cracks obviously increases. At 1200 mTorr (Figure 5c,d), it is observed the formation of well-determined nanoparticles groups, with approximately equal shapes and sizes. After 1500 mTorr (Figure 5e,f), the morphology of the films changes visibly, having a ’fluffy’ morphology, with a relatively high porosity. At 1800 mTorr and 2700 mTorr, it was observed that the films deposited, although they were very porous, they were not uniform and continuous, and these aspects eliminated from beginning the possibility of their use on the sensor. Taking into account the characteristics observed above, but also the need to highlight the influence of the porosity of the films on the quality of the sensor responses, for the manufacturing of the sensors, three deposition pressures were chosen: 100, 1200 and 1500 mTorr.

### 3.2. Sensor Properties

**Figure 6:**
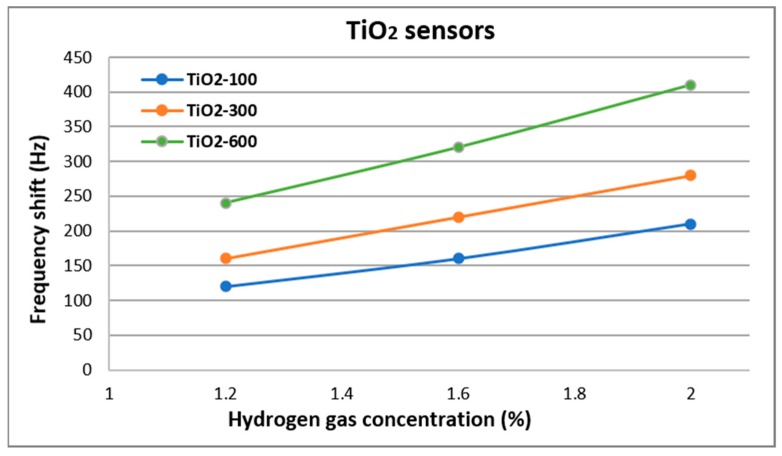
Frequency shift of the TiO_2_ sensors at different hydrogen concentrations.

**Figure 7:**
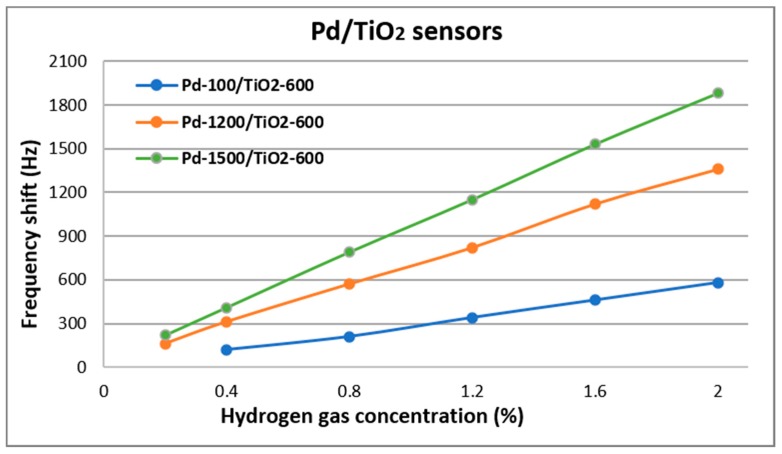
Frequency shift of the Pd/TiO_2_ sensors at different hydrogen concentrations.

Both monolayer and bilayer SAW sensors have been tested at different hydrogen concentrations at RT. The results of the frequency shifts obtained are presented in Figure 6 and Figure 7. Sensors tests were performed up to a maximum concentration of 2%, for safety reasons (hydrogen is explosive at a minimum concentration of 4%).

Regarding the sensors with TiO_2_ sensitive films, it can be seen in Figure 6 that the frequency shift increases with the increases of hydrogen concentration. As discussed above, the films deposited at different pressures have different morphology and this aspect is reflected in the sensor responses. The sensor with TiO_2_ sensitive film deposited at 100 mTorr (TiO_2_-100) was dense and it had the weakest response. As the deposition pressure increases to 600 mTorr, thus increasing the porosity of the film, the frequency shift of the sensor tested under the same conditions and at the same concentrations doubles. Thus, it can be stated that the porous morphology of the TiO_2_ sensitive film allows that hydrogen gas molecules penetrate in greater quantity the volume of the film. The mass accumulation takes place through a mechanism that influences the film properties from an electrical point of view. Negatively charged oxygen species are adsorbed on the surface of the nanostructured TiO_2_ film and they react with hydrogen reducing gas, suppling electrons in the conduction band and leading to a decrease in electric resistance [30]. Considering that these reactions take place on the surface of the film, the larger the surface of the film, the better the sensor results. 

Taking into account that for sensors with sensitive TiO_2_ films, the best result was obtained for the one deposited at 600 mTorr, for this one, the study was continued in order to improve the sensitivity. 

Through the acusto-electrical interactions, the surface acoustic wave is very sensitive at changes in electrical conductivity [37], and these interactions could be enhanced in bilayers [12]. The acusto-electrical effect comes from the interaction of electrical potential that accompanies the SAW propagation with the mobile charges in the sensing film [12]. The variation of the electrical conductivity can lead both to a decrease and to an increase of the center frequency [38]. It is known that mass accumulation of the gas leads to a decrease in the center frequency of the SAW sensor. [29]. Considering that hydrogen is a reducing gas which by reaction with the species adsorbed of oxygen releases electrons into TiO_2_ film, the conductivity increases, and the center frequency decreases. This is the mechanism that applies to the sensors studied in this work. 

Thus, to improve the sensitivity of the TiO_2_-600 sensor at RT, a bilayer of TiO_2_ and Pd was used. Because Pd has the ability to dissociate hydrogen molecules into H^+^ ions and electrons, the acusto-electrical effect between it and the TiO_2_ film is more pronounced. At the same time, the development of this process on a larger surface also leads to the improvement of the sensitivity of the sensors. This was demonstrated by sensor tests, as it is shown in Figure 7. All sensors with bilayer films (Pd/TiO_2_) showed an obvious improvement (about 3–4 times higher) of the sensitivity towards the sensors with TiO_2_ films. The measurements could be made up to a minimum concentration of 0.2%, compared to 1.2% at the sensors with TiO_2_ films. For all measurements, the sensors indicated reversibility by returning to the initial frequency after hydrogen removal. Three different morphologies of Pd were chosen to observe how the porosity of the film influences the sensor response (these were discussed at 2.1 depending on the PLD deposition pressure). As expected, for the dense Pd film, the sensor results were weaker, but better than TiO_2_ only sensor. With increasing Pd pressure deposition, the sensor response is noticeably better and about 4 times higher than the TiO_2_ only sensors. This is due to the porosity of the films, both TiO_2_ and Pd. Having a porous morphology, in the Pd films the process of dissociation of the hydrogen molecules takes place on a larger surface, and the penetrations of the H^+^ ions in TiO_2_ is faster. In this way, the mass effect and the acusto-electric effect are combined and lead to obtaining a sensor with good sensitivity at RT. It was observed that these aspects also influence the response times of the sensors, which varied between 15–45 s for hydrogen concentrations between 0.2 and 2%.

Table 2 presents the sensitivity and limit of detection (LOD) of the SAW sensors investigated in this work. Sensitivity is defined as the frequency shift in Hz per unit analyte concentration in ppm, and was determined from an average sensitivity values of the determinations at each gas concentration [13,29]. LOD is defined as three times the noise level divided by the sensitivity [13,30]. The noise level was estimated at 40 Hz. It can be seen that the best results were obtained by the sensor that had the highest porosity (Pd-1500/TiO_2_-600). 

## 4. Conclusions

The films of TiO_2_, Pd and Pd/TiO_2_ were deposited by PLD method at different oxygen and argon pressures in chamber deposition. For each pressure deposition used and depending on the material, different morphologies of the films were obtained, which were analyzed by SEM images. It has been found that with increasing deposition pressure, the morphology of the films gains a higher porosity. SAW sensors were realized at different degrees of porosity, and the tests at RT indicated that the best results were obtained through the bilayer film sensor (Pd-1500/TiO_2_-600), deposited at the highest pressures: 600 mTorr for TiO_2_ and 1500 mTorr. This sensor had a frequency shift of 1.8 kHz at 2% hydrogen concentration, a sensitivity of 0.10 Hz/ppm and a limit of detection (LOD) of 1210 ppm. For SAW gas sensors, porosity and multilayer films are properties that allow detection at RT, and PLD is a method that allows them to be obtained in an easy way, by varying the gas pressure in the deposition chamber. 

## Figures and Tables

**Table 1:** Sensors and the pressure deposition of the sensitive films.

Sensor Name	Composition of the Sensitive Layer	Deposition Pressure (mTorr)	
		O_2_	Ar
TiO_2_-100	TiO_2_	100	-
TiO_2_-300		300	-
TiO_2_-600		600	-
Pd-100/TiO_2_-600	Pd/TiO_2_	600	100
Pd-1200/TiO_2_-600		600	1200
Pd-1500/TiO_2_-600		600	1500

**Table 2:** Sensitivity and limit of detection (LOD) of the sensors. Legend: Δf-frequency change; c-hydrogen concentration, *n*-noise level.

Sensor	Sensitivity (Δf/c) (Hz/ppm)	LOD (3 × *n*)/(Δf/c) (ppm)
TiO_2_-100	0.01	11807
TiO_2_-300	0.01	8765
TiO_2_-600	0.02	5951
Pd-100/TiO_2_-600	0.02	4224
Pd-1200/TiO_2_-600	0.07	1661
Pd-1500/TiO_2_-600	0.10	1210
